# Une complication rare des léiomyomes utérins: hémopéritoine massif par rupture de varices

**DOI:** 10.11604/pamj.2013.14.110.2471

**Published:** 2013-03-21

**Authors:** Florent Fouelifack Ymele, Jovanny Fouogue Tsuala, Jeanne Hortence Fouedjio, Charlette Nangué, Caroline Kayo De Kayo, Pisoh Walter Dobgima, Robinson Enoh Mbu

**Affiliations:** 1 Hôpital Central de Yaoundé, Cameroun; 2 Groupe Associatif pour la Recherche, L'Education et la Santé GARES-Falaise, Dschang, Cameroun; 3 Département de Gynécologie et Obstétrique, Faculté de Médecine et des Sciences Biomédicales de l'Université de Yaoundé I–, Cameroun

**Keywords:** Léiomyomes utérins, hémopéritoine, varices, fibromes, uterine leiomyomas, hemoperitoneum, varices, fibroids

## Abstract

Les léiomyomes utérins sont des causes exceptionnelles d'hémopéritoine. Nous rapportons ici le cas d'une femme de 46 ans nullipare, en instance d'une hystérectomie totale indiquée pour utérus polymyomateux symptomatique. Elle a été opérée en urgence pour hémopéritoine aigu et massif compliqué de choc hémorragique. L'origine de l'hémopéritoine était la rupture spontanée d'une varice du léiomyome. Quoique rare l'éventualité d'un hémopéritoine causé par un fibrome utérin devrait être évoquée devant tout abdomen aigu spontané chez une femme en âge de procréer. La présence de varices sur les fibromes augmenterait le risque d'hémorragie spontanée.

## Introduction

Communément appelés fibromes, les léiomyomes utérins sont les tumeurs les plus fréquentes de la femme pendant la vie reproductive. Ils sont asymptomatiques dans 50% des cas [1]. Les symptômes les plus fréquents sont les saignements utérins anormaux et les manifestations liées à la compression des organes voisins (pollakiurie et constipation). L'hémopéritoine causé par un myome reste exceptionnel [2]. Nous présentons le cas d'une femme de 46 ans reçue et prise en charge en urgence dans l'Unité de Gynécologie et Obstétrique de l'Hôpital Central de Yaoundé pour léiomyomes utérins compliqués d'hémopéritoine massive.

## Patient et observation

Il s'agissait de Mme NNJJ âgée de 46 ans, nullipare, célibataire, pasteur dans une église de la ville de Yaoundé, amenée en urgence pour douleur abdominale aigue généralisée survenue spontanément deux heures avant la consultation, après un passage brusque à la position assise. L'évolution était marquée par l'installation rapide d'une asthénie associée à des vertiges, une soif intense et une augmentation progressive du volume de l'abdomen. La patiente était déjà suivie dans notre service pour un volumineux utérus polymyomateux symptomatique pour lequel une hystérectomie totale était programmée quatre jours plus tard. Comme antécédents, elle souffrait d'une infertilité primaire depuis 26 ans. Elle souffrait de ménométrorragies depuis 16 ans, attribuées à son utérus polymyomateux pour lequel elle avait refusé jusque-là tout traitement chirurgical, préférant plutôt les traitements par la prière et les naturopathes. Aucune méthode contraceptive n'était utilisée par la patiente. Elle était de groupe sanguin B rhésus positif et souffrait d'une anémie chronique bien tolérée. Elle n'avait jamais reçu de transfusion sanguine. A l'enquête des systèmes, outre le motif de consultation, la patiente avait soif, des palpitations, une sensation de froid et une dyspnée. Elle était asthénique et n'avait ni de fièvre, ni d'arrêt de matière et de gaz.

A l'examen physique, la patiente avait un état général altéré. Elle était consciente et orientée dans le temps et dans l'espace. Le pouls était de 120 pulsations par minute, la pression artérielle de 100/60 millimètres de mercure, la fréquence respiratoire de 36 cycles par minute, et la température de 37,1degrés Celsius. A l'inspection, les conjonctives étaient très pâles, l'abdomen distendu, et la patiente couverte de sueur. On notait à la palpation une défense abdominale diffuse et pas de contracture. On palpait une masse polylobée abdominopelvienne sensible indissociable de l'utérus, de 31 centimètres au-dessus de la symphyse pubienne et de 26 centimètres dans le plan axial. Par ailleurs, on notait une matité déclive des flancs et un refroidissement des extrémités. La circonférence abdominale à hauteur de l'ombilic était de 98 centimètres. Au toucher vaginal le col était long et fermé, les culs-de-sac vaginaux bombés et peu sensibles. La masse était mobile avec le col. Les annexes utérines n'étaient pas appréciables du fait de la distension abdominale.

Devant cette symptomatologie, nous avons pensé à un hémopéritoine avec pour étiologies probables: une grossesse extra utérine rompue, un kyste ovarien rompu, la rupture de varices d'un léiomyome, la rupture du pédicule d'un myome sous-séreux pédiculé ou la rupture d'un organe plein. La paracentèse faite a ramené 10 millilitres de sang non coagulable, confirmant ainsi la présence d'un hémopéritoine. La négativité du test de grossesse urinaire nous a permis d'exclure l'hypothèse d'une grossesse extra-utérine rompue. Avec l'accord de l'anesthésiste-réanimateur et du chirurgien, l'indication d'une laparotomie exploratrice était posée en urgence, le « counseling » préopératoire fait et une réanimation préopératoire immédiatement entreprise par deux voies veineuses centrales. Elle a été mise sous oxygène au masque, 2000 millilitres (soit 4 unités) de sang total ont été requis et l'ordonnance préopératoire remise à la famille. La patiente est arrivée avec un bilan préopératoire dont les résultats étaient les suivants: taux d'hémoglobine 9,7 grammes par décilitre, hématocrite à 31,9%, taux de globules blancs 6400 par millimètre cube de sang, taux de plaquettes 217000 par millimètre cube de sang, volume globulaire moyen de 76 millimètres cube, teneur globulaire moyenne en hémoglobine de 23 picogrammes, concentration corpusculaire moyenne en hémoglobine de 30,4 grammes par décilitre, bilan de coagulation normal, INR égale à 1, glycémie à jeun à 0,74 gramme par litre de sang, créatininémie à 8,9 milligrammes par litre de sang, urémie à 0,18 grammes par litre de sang. L'échographie réalisée 2 mois avant l'admission aux urgences montrait un gros utérus contenant 5 noyaux myomateux interstitiels corporeals fundiques et un sous séreux fundique à base sessile.

**Figure 1:**
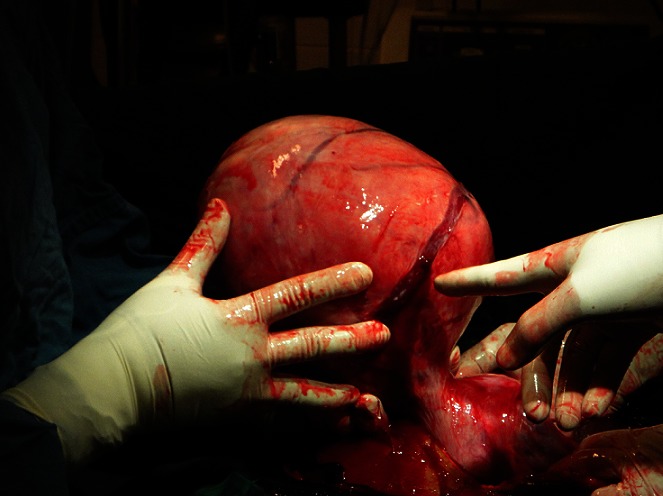
Trouvailles per opératoires de la laparotomie indiquée pour hémopéritoine sur léiomyomes utérins. On observe un gros léiomyome sous séreux pédiculé, avec du sang jaillissant du site de rupture de la varice.

**Figure 2:**
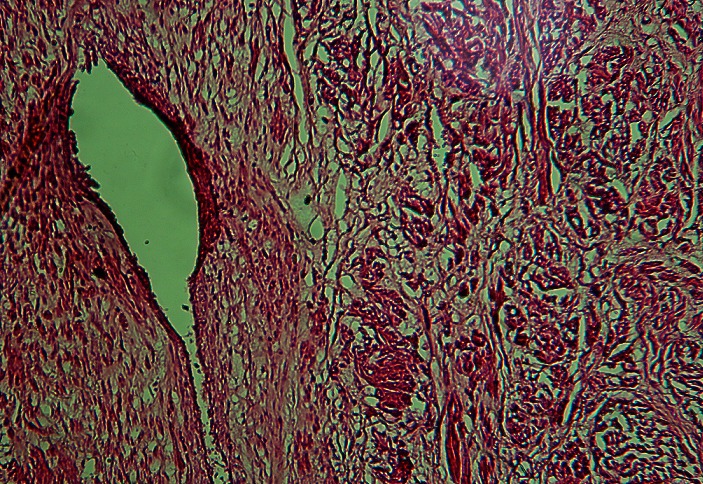
Planche montrant une coupe au microtome après coloration à l'hématoxiline-éosine au grossissement 100× du léiomyome. On y voit les fibres musculaires lisses et un gros vaisseau irrigant le léiomyome

La laparotomie était réalisée six heures plus tard après l'admission, par abord médian sous et sus ombilicale contournant l'ombilic par la gauche. Les trouvailles étaient un hémopéritoine de 2500 millilitres, un utérus polymyomateux dont le plus gros, sous séreux sessile avait un diamètre de 22 centimètres et présentait de nombreuses varices dont la plus large (environ 10 millimètres de diamètre) était rompue et saignait activement (Figure 1). Les annexes utérines étaient macroscopiquement normales, et le pelvis sans adhérences. Une hystérectomie totale inter-annexielle a été réalisée et le poids total de la pièce était de 5500 grammes. Mille millilitres de sang total ont été transfusées en per opératoire et 500 millilitres en post opératoire immédiat. Les suites opératoires étaient simples et la patiente sortie de l'hôpital au septième jour postopératoire. L'analyse anatomopathologique de la pièce opératoire a conclu à un léiomyomatose utérine très vascularisée (Figure 2) sans signes de malignité.

## Discussion

En dehors de la grossesse, les symptômes des léiomyomes incluent: métrorragies, ménorragies, dysménorrhée, distension abdominale disgracieuse et pelviagies non menstruelles [1]. D'autres symptômes sont liés à la compression des organes avoisinants. Au moment du diagnostic de ses léiomyomes notre patiente présentait des ménorragies et une importante distension abdominale. Du point de vue obstétrical les léiomyomes utérins seraient responsables de subfertilité, d'avortements, d'accouchements prématurés, de mal présentations f'tales et d'hémorragies du post-partum. Notre malade souffrait d'infertilité primaire. L'incidence et taille des myomes sont plus élevées dans la race noire à laquelle appartenait notre patiente. La prévalence des fibromes symptomatiques est maximale en péri ménopause et notre patiente était âgée de 46 ans [1].

L'hémopéritoine causé par des fibromes utérins reste extrêmement rare. Le cas le plus ancien a été rapporté dans la littérature en 1931[2]. Le cas que nous présentons est le premier à être rapporté au Cameroun. La rareté et la gravité de ce tableau expliquent le fait que le diagnostic étiologique soit presque toujours posé en per opératoire ou au cours de l'autopsie lorsque les fibromes ne sont pas connus d'avance. Les diagnostics différentiels conduisant à la laparotomie sont: la rupture ou l'abrasion d'un cancer ovarien, une grossesse extra-utérine rompue, la rupture de la rate ou un kyste ovarien hémorragique [3–5]. Dans notre cas le lien entre l'hémopéritoine et les myomes a été suspecté car la malade était en attente d'une hystérectomie indiquée pour gros utérus polymyomateux symptomatique.

Les mécanismes physiopathologiques de l'hémopéritoine due aux fibromes sont les suivants: rupture d'un fibrome, avulsion d'un myome sous séreux pédiculé, et rupture d'artères ou de veines avoisinant ou irriguant ou drainant le fibrome. Le caractère variqueux des veines périmyomateuses fragilise ces dernières, qui constituent ainsi un facteur de risque de rupture et donc d'hémopéritoine [6]. Dans notre cas il s'agissait d'une rupture d'une grosse veine variqueuse drainant le léiomyome. La taille du fibrome est corrélée à ses besoins nutritifs. Ainsi de plus gros fibromes sous séreux présentent plus fréquemment une hyper vascularisation artérielle et veineuse. Le fibrome porteur de la varice rompue chez notre malade avait un diamètre de 22 centimètres.

Dans certains cas l'hémopéritoine est déclenché par des contusions abdominales ou par une hyperpression abdominale. Cette hyperpression abdominale serait le facteur déclenchant de la rupture de la varice chez notre patiente car dans l'histoire, la douleur a commencé juste après qu'elle se soit assise brusquement. Le volume de l'hémopéritoine dépendrait de la nature et du nombre de vaisseaux rompus, du délai de prise en charge, et du colmatage éventuel de la brèche vasculaire par un organe avoisinant. Nous avons trouvé un hémopéritoine de 2500 millilitres sur une rupture d'une varice d'environ 1 centimètre de diamètre, saignant activement. Le long délai de prise en charge de notre patiente (six heures entre son accueil aux urgences et le début de la chirurgie) découle du fait que nous n'avions pas de kit d'urgence ou de sang à notre disposition. Malgré l'urgence, la famille a fait le tour des pharmacies de la ville et des banques de sang des hôpitaux de la ville pour ramener les médicaments et 3 unités (1500 millilitres) de sang transfusées en per et post opératoire. Ceci rappelle la problématique du retard de prise en charge des urgences dans notre milieu aux ressources limitées [7, 8].

## Conclusion

La réalité et la gravité de l'hémopéritoine causé par les léiomyomes utérins ne doivent pas être occultées dans l'esprit du praticien par son extrême rareté. L'intérêt de ce cas est d'attirer l'attention du clinicien sur la nécessité de penser à l'hémopéritoine chez les patientes porteuses de myomes et qui présentent une symptomatologie pareille.
